# Association of the platelet-to-albumin ratio with diabetic nephropathy lesions via a fine-tuning-free large language model framework

**DOI:** 10.3389/fmed.2026.1793422

**Published:** 2026-05-20

**Authors:** Wenbo Xia, Dongyang Shen, Jian Chen, Ting Liang, Mei Wang, Yongcai Gao, Bo Li, Yali Zheng

**Affiliations:** 1 People's Hospital of Ningxia Hui Autonomous Region, Ningxia Medical University, Yinchuan, China; 2 Ningxia Research Institute of Transport Sciences, Yinchuan, China; 3 The First People's Hospital of Shizuishan, Shizuishan, China

**Keywords:** diabetic nephropathy, large language models, machine learning, non-invasive diagnosis, platelet-to-albumin ratio

## Abstract

**Objective:**

The current research aims to utilize a fine-tuning-free, freely available large language model (LLM) framework to systematically investigate the correlation between the platelet-to-albumin ratio (PAR) and the pathological severity of diabetic nephropathy (DN). This approach aims to develop an exploratory risk stratification tool for this microvascular complication.

**Methods:**

A retrospective analysis was conducted on 195 patients diagnosed with DN. The effectiveness of the PAR as a diagnostic tool was evaluated. A “tabular-to-text-to-vector” framework was developed, utilizing the frozen Qwen and Llama models to extract semantic features from the serialized clinical narratives. The performance of this model was compared with traditional algorithms using a 5-fold cross-validation technique.

**Results:**

Receiver operating characteristic (ROC) analysis of the current data indicated that the optimal cutoff for the PAR was 7.155, with an area under the curve (AUC) of 0.716. Multivariate logistic regression analysis revealed a positive correlation between PAR levels and the pathological severity of DN (OR: 6.65, 95% CI: 2.617–16.9). The fusion LLM framework showed improved balance in addressing class imbalance achieving higher specificity (56.67%) compared to the random forest (RF) model (31.67%), although improvements in overall AUC were marginal. Regarding the assessment of interstitial fibrosis and tubular atrophy (IFTA), the model's macro-F1 score of 51.00 ± 5.71%, exceeded that of the XGBoost model, which recorded a score of 45.22%.

**Conclusion:**

The platelet-to-albumin ratio was significantly associated with the pathological severity of DN. As an exploratory proof-of-concept, the fine-tuning-free fusion LLM framework proposed in this study utilizes semantic reasoning and demonstrates potential scalability for small-sample medical datasets, particularly in non-invasive risk stratification.

## 1 Introduction

Diabetic nephropathy (DN) is the foremost cause of end-stage renal disease (ESRD) around the world (1, 2). A thorough assessment of renal pathology is essential for informing prognosis and guiding treatment decisions (3). The classification established by Renal Pathology Society (4) is recognized as the gold standard for evaluating DN lesions and employs glomerular classification and interstitial fibrosis and tubular atrophy (IFTA) scoring to guide renal biopsies (5). Given that clinical and pathological factors alone are inadequate for reliably forecasting the kidney outcomes for patients with DN, the development of non-invasive biomarkers (6) and computational models to stratify DN patients based on varying levels of renal risk could offer more precise guidance for treatment and enhance prognostic outcomes.

The platelet-to-albumin ratio (PAR) is an emerging biomarker that may reflect systemic inflammatory and metabolic changes (7). However, only a limited number of studies have demonstrated a precise correlation between platelet-associated parameters and the severity of DN pathology. An elevated platelet count (PLT) can indicate a pro-inflammatory and hypercoagulable state, which may exacerbate glomerular endothelial injury (8–10). Conversely, hypoalbuminemia in patients with DN serves as an indicator of malnutrition or severe proteinuria, both of which are well-established contributors to renal deterioration (11, 12). By integrating various physiological signals, the PAR facilitates a more comprehensive evaluation of the inflammation-nutrition status than any single parameter could provide (13). Utilizing biomarkers such as PAR to elucidate complex and non-linear relationships within multidimensional clinical data presents significant challenges, particularly when performing analyses in biopsy-confirmed cohorts characterized by small sample sizes (14, 15).

Conventional machine learning algorithms, such as random forest (RF) and XGBoost models, often struggle to address these data-related issues (16). These models typically treat clinical indicators as mere numerical values, disregarding their semantic context and the intricate relationships between these indicators (17). For example, RF models tend to be highly sensitive; however, they often lack specificity, which can result in overdiagnosis during clinical screenings (18). Large language models (LLMs) have revolutionized the fields of biomedical and health informatics with their powerful ability to interpret natural language (19, 20). In the domain of occupational health informatics, previous studies have introduced the “Weaving Embeddings” framework (21, 22). This lightweight fusion architecture does not require fine-tuning; instead, it combines various semantic embeddings from off-the-shelf LLMs such as Qwen and Llama (23, 24). By leveraging a co-occurrence strategy for second-order interactions, LLMs achieve state-of-the-art accuracy in classifying unstructured text while demonstrating greater computational efficiency compared to fine-tuned baselines. Building upon the principles of efficient unstructured data processing with limited resources, this paradigm of fine-tuning-free LLM fusion is hypothesized to adapt effectively to structured clinical datasets (25, 26).

The current work proposes a “tabular-to-text-to-vector” framework for non-invasive grading of DN, aiming to bridge structured tabular data with the linguistic capabilities of LLMs. This method leverages inherent medical knowledge embedded within pretrained model architectures for generating narratives from discrete clinical variables such as the PAR, estimated glomerular filtration rate (eGFR), and proteinuria (27). By incorporating features from heterogeneous LLMs, the proposed architecture can address limitations associated with small sample sizes and complex feature spaces (28). This study elucidates the relationship between the PAR and the pathological severity of diabetic nephropathy. The findings indicate that aggregating semantic reasoning from various models outperforms conventional baselines, even in data-limited contexts, thereby providing a scalable and efficient solution for clinical nephrology.

## 2 Methods

### 2.1 Patient selection

This retrospective study evaluated individuals with DN who underwent renal biopsy at the People's Hospital of Ningxia Hui Autonomous Region from May 2018 to June 2025. A total of 257 cases were screened, and DN was diagnosed based on the 2010 DN classification system. Renal biopsies were performed when patients presented with clinical indications requiring pathological clarification, including: (1) rapid decline in renal function of unknown cause; (2) significant proteinuria (≥0.5 g/day) without diabetic retinopathy; (3) presence of active urinary sediment (e.g., hematuria or cellular casts); or (4) clinical suspicion of coexisting non-diabetic renal disease. These criteria were applied to identify a specific subset of DN patients with atypical or severe presentations, as renal biopsy remains essential for differentiating diabetic nephropathy from superimposed glomerular diseases that may necessitate divergent management strategies. The following exclusion criteria were applied: inadequate pathological specimens (defined as insufficient data or fewer than 10 glomeruli in renal biopsy), presence of other types of diabetes mellitus, non-diabetic nephropathy, unclear medical history, incomplete clinical data, eGFR less than 30 mL/min/1.73 m^2^, active infection at enrollment, or prior use of prednisone or other immunosuppressive therapies. These criteria were established to avoid confounding effects on the platelet-related parameters. After the implementation of these exclusion criteria, the final analysis included 195 patients. This research was authorized by the Ethics Committee of the People's Hospital of Ningxia Hui Autonomous Region and conducted in line with the Declaration of Helsinki. The requirement for informed consent was waived due to the retrospective nature of the study.

### 2.2 Clinical and histopathological assessment

Included in the clinical characteristics of the patients were demographic data such as sex, age, and body mass index (BMI). Additionally, the patients' history of diabetes, including type and duration, as well as fasting blood glucose (FBG), were documented. Laboratory parameters such as 24-h proteinuria, serum albumin, serum creatinine (sCr), blood urea nitrogen, uric acid, hemoglobin (Hb), PLT, triglycerides, low-density lipoprotein (LDL), and high-density lipoprotein (HDL) were also extracted from the electronic medical records. Furthermore, the presence of hypertension was noted during the documentation process. However, specific data regarding the usage of Renin-Angiotensin-Aldosterone System (RAAS) inhibitors (ACEIs/ARBs) were not available in the retrospective records and thus could not be included in the analysis.

The eGFR was determined using the Chronic Kidney Disease Epidemiology Collaboration (CKD-EPI) formula (29). All renal biopsies underwent light microscopy, immunofluorescence, and electron microscopy. According to the 2010 Renal Pathology Society classification system, DN was pathologically categorized into classes I–IV based on glomerular lesions, while IFTA were graded on a four-point scale ranging from 0 to 3 (4). For the predictive modeling task, IFTA scores were re-categorized into three classes: Mild (scores 0–1), Moderate (score 2), and Severe (score 3).

### 2.3 Statistical analysis

Missing data were handled via complete-case analysis; patients with missing values for key clinical variables were excluded during the screening process, resulting in a final analytic cohort of 195 patients with complete data for all included. The Mann-Whitney U-test was employed to evaluate differences between the two groups. ROC curve analysis was performed to derive an exploratory threshold of serum PAR levels for identifying the severity of renal pathology at the time of kidney biopsy in patients with diabetes, based on the maximization of the Youden index. This study employed a retrospective cross-sectional design to investigate the association between serum PAR levels at the time of renal biopsy and the severity of renal pathological changes in patients with DN. Key clinical confounders, namely age, sex, blood pressure, and serum C-reactive protein (CRP) level, along with covariates that were statistically significant in the univariate analysis, were entered into the multivariable logistic regression model. Given the structural collinearity between PAR and its components (platelet count and albumin), the primary multivariable model included PAR while excluding platelet count and albumin. This approach was chosen to assess the independent predictive value of the composite index. Sensitivity analyses were performed by separately including platelet count and albumin in alternative models to evaluate their individual contributions. To evaluate potential non-linear relationships, restricted cubic splines (RCS) with three knots were used to model PAR in the multivariable logistic regression. A likelihood ratio test was performed to assess non-linearity. A *P-value* of less than 0.05 was deemed statistically significant in this study. Statistical analyses were conducted using GraphPad Prism and SPSS Statistics version 24.0 (SPSS Inc., Chicago, IL, USA).

### 2.4 Fusion LLM methodology

**Figure 1:**
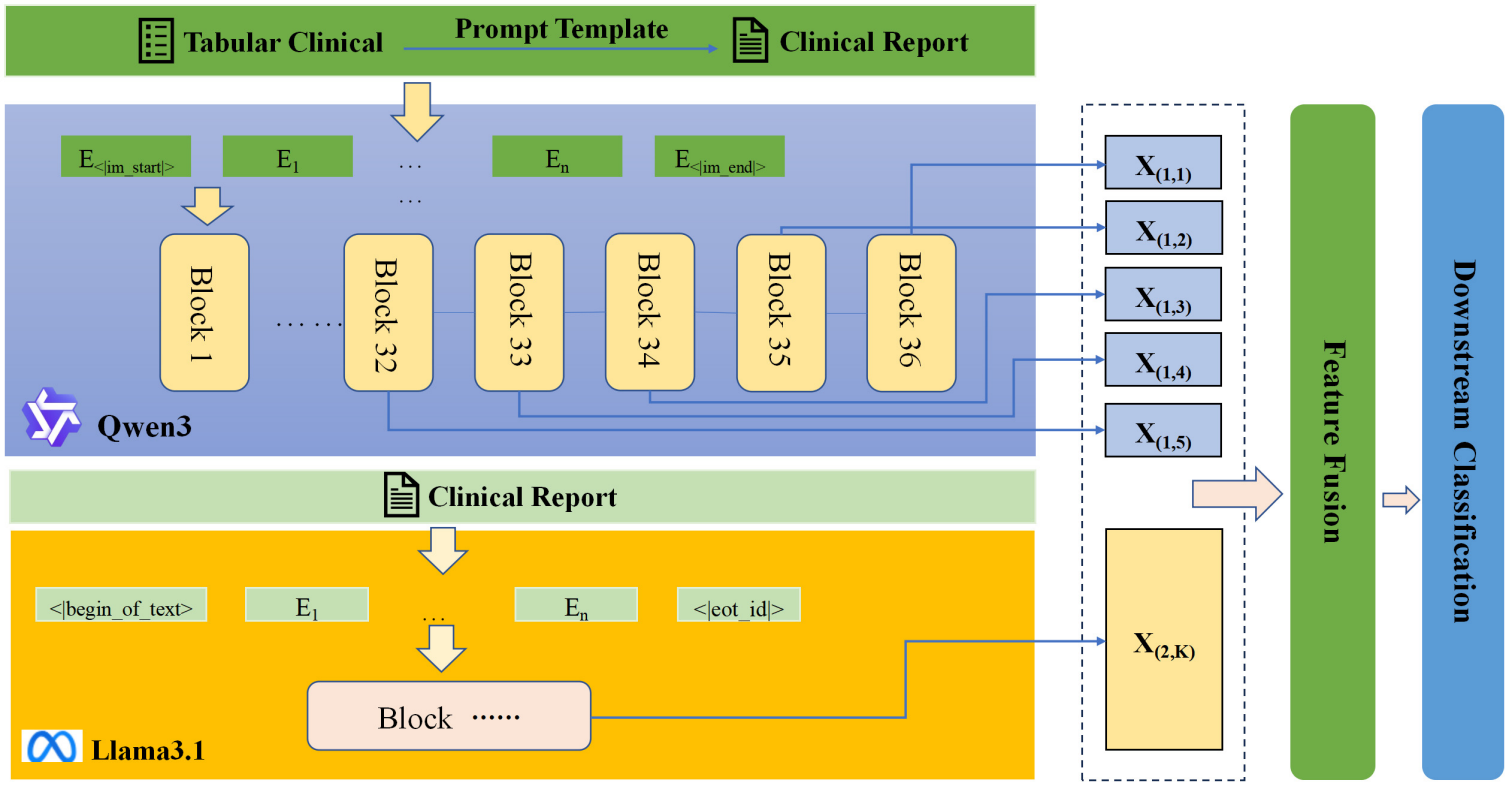
Architecture of the proposed “tabular-to-text-to-vector” fusion framework for noninvasive grading of DN.

The goal of the current investigation is to develop a novel framework termed “tabular-to-text-to-vector” aimed at addressing challenges associated with small sample sizes and high-dimensional features in non-invasive grading of DN. This method convert's structured clinical tabular data into natural language, thereby leveraging reasoning capabilities of LLMs. Two pre-trained LLMs, Qwen3-8B and Llama3.1-8B-Instruct, were used to extract high-dimensional semantic features which were fused to train a light-weight downstream classifier. The overall architecture is illustrated in Figure 1.

#### 2.4.1 Data serialization: from tabular to text

Traditional machine learning models often treat clinical indicators as independent variables, neglecting interrelationships among them. To address this limitation, a serialization process for data has been implemented. $Let\;D\;=\left\{\left(\;x_{i},y_{i}\;\right)\right\}\frac{N}{i=1}$ denote the dataset of *N* patients; where *x*_*i*_ represents the tabular clinical features (e.g., sCr, 24-h proteinuria, and eGFR); and y_*i*_ represents the pathological label (IFTA score or glomerular lesion classification). For each patient *i*, a prompt template *P*(·) was constructed to map the numerical vector (*x*_*i*_) into a natural language clinical report (*T*_*i*_) as follows Equation 1:


$$\begin{array}{l} \text{Ti}=\text{P}\left(x_{i}\right) \end{array}$$ (1)

The exact prompt template utilized is as follows: “Basic Information: {Age} years old {Gender}, history of diabetes for {History} years. Physical Examination: BMI {BMI}, {BP_Description}. Renal Function: eGFR is {eGFR} ml/min, Serum Creatinine {SCr} umol/L. Twenty-four hour proteinuria severity is {Proteinuria} mg. Serum Albumin {ALB} g/L, BUN {BUN} mmol/L, Uric Acid {UA} umol/L. Core Indicator: Platelet-to-Albumin Ratio (PAR) is {PAR}. Metabolic & Hematologic Context: Glucose {Glu} mmol/L, Lipids (Triglycerides {TG}/Cholesterol {Chol}). Hemoglobin {Hb} g/L, CRP {CRP} mg/L, Platelets {PLT}, WBC {WBC} (Neutrophils {NE}%/Lymphocytes {LY}%).” For example, tabular data for a specific patient is directly converted into a generated narrative such as: “Basic Information: 58 years old Male, history of diabetes for 10 years. Physical Examination: BMI 26.5, Comorbid Hypertension… ” This *T*_*i*_ textual representation encapsulated patient's profile in a format that simulates a physician's diagnostic narrative, thereby activating prior medical knowledge embedded within LLMs. A comprehensive breakdown of the serialization logic, including mapping rules for binary flags (e.g., “Comorbid Hypertension”), and a complete conversion example from raw numerical data to the final textual narrative are provided in Supplementary Table S1.

#### 2.4.2 Dual-stream semantic feature extraction

A dual-stream architecture was employed to extract complementary feature representations from the generated text (*T*_*i*_). Two distinct LLMs, denoted as *LLM*_*Q*_ (Qwen) and *LLM*_*L*_ (Llama), served as fixed feature extractors. The parameters of both LLMs were frozen during the training phase.

Unlike traditional approaches that only utilize the embedding from the final layer, the different layers of LLMs may capture different levels of semantic abstraction. To preserve richer semantic information, hidden states from the last *K* = 5 transformer layers of each model were extracted. Given that both Qwen and Llama are causal language models (decoder-only architectures) employing a self-attention mechanism where each token attends only to its predecessors, the representation of the last token in the sequence aggregated the global context of the input. Therefore, a last token pooling strategy was employed. For a given input (*T*_*i*_) and a specific layer (*k*; where *k* {*L-4,...,L*} and *L* are the total number of layers), let ${\text{H}}_{i}^{\left(k\right)}\in \;R^{S\times D}$ denote the hidden states sequence, where *S* is the sequence length and *D* is the hidden dimension. The layer-specific feature vector $v_{i}^{\left(k\right)}$ was defined as follows Equation 2:


$$\begin{array}{l} v_{i}^{\left(k\right)}=\;{\text{H}}_{i}^{\left(k\right)}\left[last_{-}token_{-}idx\right] \end{array}$$ (2)

By stacking these vectors, a multilayer semantic representation tensor (**F**_***i***_ *R*^*k*×*D*^) was obtained for each model, resulting in the following two feature tensors: **F**_*Q, i*_ derived from Qwen and **F**_*L, i*_ derived from Llama.

#### 2.4.3 Feature fusion strategy

To mitigate the curse of dimensionality and the severe overfitting risk on our small cohort, we replaced straightforward concatenation with a second-order co-occurrence fusion strategy. First, the 10 hidden states $v_{j}\in R^{4096}$ were individually compressed to 1,024 dimensions via linear projections (Equation 3):


$$\begin{array}{l} u_{j}=ReLU\left(W_{j}v_{j}+b_{j}\right) \end{array}$$ (3)

These compressed vectors were stacked into a matrix *X*∈*R*^10 × 1024^ to compute a cross-layer semantic co-occurrence matrix *C* = *XX*^*T*^∈*R*^10 × 10^. This matrix was then flattened into a 100-dimensional vector *c*_*flat*_ and normalized using a trainable scaling factor γ (Equation 4):


$$\begin{array}{l} p=2\cdot Sigmoid\left(\gamma \cdot c_{flat}\right)-1 \end{array}$$ (4)

Finally, $p$ was concatenated with the mean-pooled embedding of the primary LLM $\left(v_{Q}\in R^{4096}\right)$ to form the downstream feature vector *f*_*i*_ (Equation 5):


$$f_{i}=Concat\left(p,v_{Q}\right)$$ (5)

This strategy successfully reduced the final feature space to exactly 4,196 dimensions, preserving crucial semantic interactions while substantially lowering the risk of overfitting.

#### 2.4.4 Downstream classification

The *f*_*i*_ fused feature vector was fed into a downstream multilayer perceptron (MLP) for final classification. The MLP consisted of a projection layer, a non-linear activation function, and a classification head. Specifically, the high-dimensional feature (*f*_*i*_) was projected to a lower dimensional hidden space, followed by a rectified linear unit (ReLU) activation and a dropout layer (with rate *p* = 0.5) to mitigate overfitting. The final layer mapped the hidden representation to the following output logits: z_*i*_ ε R^C^; where *C* is the number of target classes (e.g., *C* = 2 for glomerular lesion classification or *C* = 3 for IFTA scoring).

The total number of trainable parameters in the downstream architecture is approximately 46.5 million. Given the potential risk of overfitting associated with training ~46.5 million parameters on a 195 dataset, we implemented aggressive regularization strategies: (1) extensive real-time data augmentation injecting Gaussian noise scaled up to 20 times; (2) substantial dropout rates (*P* = 0.5) applied after every activation layer; (3) weight decay (1 × 10^−2^) utilizing the AdamW optimizer; and (4) label smoothing regularization (α = 0.1).

#### 2.4.5 Loss function with class balancing and smoothing

Given the inherent class imbalance in the present biopsy-proven dataset (where severe cases may outnumber mild cases or vice versa), standard cross-entropy loss may lead to biased predictions. To address this, a weighted cross-entropy loss combined with label smoothing was employed.

First, to counteract the class imbalance, class-specific weights (*w*_*c*_) inversely corresponding to the class frequencies were computed in the training set as follows Equation 6:


$$\begin{array}{l} w_{c}\;=\frac{1}{Nc\;+\;\epsilon } \end{array}$$ (6)

where *Nc* is the number of samples in class *c*, and ϵ is a small constant for numerical stability. These weights were then normalized to have a unit mean before being applied to the loss function.

Furthermore, to prevent the model from becoming overconfident and to improve generalization of the small-scale dataset, label smoothing regularization was introduced. Instead of using hard one-hot encoded targets (*y*), smoothed targets (*y*^*LS*^) were used as follows Equation 7:


$$\begin{array}{l} y_{k}^{LS}\;=\;\left(1\;-\;\alpha \right)yk\;+\frac{\alpha }{C} \end{array}$$ (7)

where *yk* is the original ground truth; *C* is the number of classes; and α = 0.1 is the smoothing parameter used in the experiments.

The final objective function was formulated as follows Equation 8:


$$\begin{array}{l} L\;=\;-\frac{1}{N}\overset{N}{\underset{i=1}{\sum }}w_{yi}\overset{c}{\underset{k=1}{\sum }}y_{i,k}^{LS}\;log\left(\frac{exp\left(z_{i,k}\right)}{\sum_{j=1}^{C}\exp\left(z_{i,j}\right)}\right) \end{array}$$ (8)

This combined loss function ensured that the model paid equal attention to minority classes while maintaining robust decision boundaries.

### 2.5 Fusion LLM experimental setup

#### 2.5.1 Implementation details

All experiments were implemented using the PyTorch framework on a single RTX 4090 NVIDIA GPU. The proposed fusion LLM framework was trained in an end-to-end manner. For the optimization, the AdamW optimizer, with a weight decay of 1 × 10^−2^, was utilized to regularize the model parameters. The initial learning rate was set to 1 × 10^−4^, coupled with a cosine annealing scheduler to dynamically adjust the learning rate over 300 epochs, ensuring stable convergence.

To accommodate the small sample size (*N* = 195) and prevent overfitting, a batch size of 32 was employed. Regarding data preprocessing, since the final analytic cohort had no missing values (complete-case analysis), no imputation was required. Prior to training the baseline models, all numerical tabular features underwent standard scaling, with the scaler similarly fitted solely on the training folds. The baseline models were configured with robust parameters to ensure optimal performance, such as using a radial basis function (RBF) kernel for support vector machine (SVM), 100 estimators for Random Forest, and a maximum of 1,000 iterations for Logistic Regression. As detailed in the Methodology section, a weighted cross-entropy loss was utilized to address class imbalance, with class weights dynamically calculated based on the inverse frequency of each class in the training fold. Additionally, label smoothing with a factor of α = 0.1 was applied to all classification tasks to prevent the model from becoming overconfident and to improve generalization on unseen data.

#### 2.5.2 Evaluation metrics

The model performance was evaluated using a rigorous 5-fold stratified cross-validation strategy to ensure that the class distribution in each fold remained consistent with the overall dataset, providing a reliable estimate of the generalization capability of the model. For the performance assessment, the following metrics were employed: (1) F1-Score, which represents the harmonic mean of precision and recall—the macro-F1 score was reported for the 3-class IFTA prediction to treat all classes equally, and the binary F1 score was reported for the 2-class glomerular lesion classification for the positive class (severe type); (2) area under the ROC curve (AUC), which evaluates the ranking ability and discriminatory power of the model across different decision thresholds; (3) sensitivity (recall), which measures the proportion of true positive cases correctly identified—this is crucial for minimizing missed diagnoses in clinical screening; and (4) specificity, which measures the proportion of true negative cases correctly excluded, reflecting the ability of the model to avoid false alarms.

#### 2.5.3 Baselines

To benchmark the effectiveness of the proposed method, it was compared against the following widely used machine learning algorithms trained on the original numerical tabular data: a clinical baseline model using logistic regression (LR) incorporating only the four core clinical variables: age, eGFR, 24-h proteinuria, and PAR (continuous variable), as well as traditional machine learning algorithms including logistic regression (LR) (30), which is a linear baseline to assess the linearity of the features; SVM (31), which utilizes a RBF kernel, effective for small-sample high-dimensional data; RF (32) , which is an ensemble method known for its robustness and high sensitivity; and XGBoost, which is a state-of-the-art gradient boosting framework in traditional tabular data processing (33).

## 3 Results

### 3.1 Comparison of clinicopathological characteristics between DN patients with different glomerular lesion severities

Patients were categorized into two groups based on the severity of glomerular lesions according to the 2010 Renal Pathology Society classification: class I–II (mild) and class III–IV (severe). This study enrolled 195 patients diagnosed with diabetic nephropathy based on kidney biopsy findings. The clinicopathological characteristics at diagnosis are summarized in Table 1. The cohort consisted predominantly of males (71.7%) with a median age of 52 years. Patients with severe glomerular lesions exhibited significantly higher PLT counts, sCr, 24-h proteinuria, and PAR compared to those with mild glomerular lesions. Conversely, they demonstrated significantly lower Hb levels and eGFR.

**Table 1:** Clinical pathological characteristics of all patients.

Clinical characteristics	Diabetic nephropathy			
	All patients	I-II	III-IV	*P-value*
Sex				
Male (%)	140 (71.79)	28 (14.36)	112 (57.43)	1.00
Female (%)	55 (28.21)	11 (5.64)	44 (22.56)	
**Age (years)**	52 (45, 60)	53 (40, 62)	52 (45, 60)	0.939
Hypertension				
Absent (%)	57 (29.23)	15 (7.69)	42 (21.54)	0.156
Present (%)	138 (70.77)	24 (12.31)	114 (58.46)	
**BMI (kg/m** ^**2**^ **)**	24.77 (22.66, 26.90)	25.34 (23.95, 27.22)	24.66 (22.49, 26.88)	0.280
**WBC (10** ^**9**^ **/L)**	6.24 (5.30, 7.88)	6.32 (5.51, 7.83)	6.20 (5.30, 7.95)	0.708
**NE (%)**	60.30 (54.60, 67.40)	61.0 (55.6, 67.1)	60.3 (54.2, 67.85)	0.743
**LY (%)**	28.30 (21.60, 33.10)	27.5 (21.3, 32.3)	28.5 (21.7, 33.58)	0.476
**RBC (10** ^**12**^ **/L)**	4.21 (3.53, 4.73)	4.73 (4.22, 5.12)	4.09 (3.44, 4.57)	0.487
**Hb (g/L)**	125.0 (106.0, 142.0)	142.0 (125.0, 154.0)	122.5 (105.0, 135.0)	**0.008**
**PLT (10** ^**9**^ **/L)**	238.0 (203, 273)	215 (200, 248)	246 (204.25, 281.50)	**0.004**
**ALB (g/L)**	30.81 ± 5.54	32.31 ± 3.95	30.43 ± 5.83	0.060
**CRP (mg/L)**	2.10 (1.27, 3.12)	2.03 (0.75, 3.15)	2.10 (1.28, 3.12)	0.186
**TG (mmol/L)**	1.60 (1.12, 2.32)	1.83 (1.16, 2.54)	1.57 (1.10, 2.22)	0.294
**Chol (mmol/L)**	5.03 (4.20, 5.97)	4.38 (3.95, 5.57)	5.11 (4.24, 6.05)	0.406
**Glu (mmol/L)**	6.70 (5.03, 9.76)	7.20 (5.27, 10.35)	6.63 (4.85, 9.32)	0.677
**BUN (mmol/L)**	7.60 (5.68, 10.15)	6.80 (4.92, 9.69)	7.78 (5.91, 10.56)	0.134
**UA (μmol/L)**	343 (291, 397)	329 (280, 395)	344 (292, 400)	0.701
**sCr (μmol/L)**	104.70 (76.40, 145.00)	85.7 (68.0, 112.0)	110.75 (78.03, 152.95)	**< 0.007**
**eGFR (ml/min/1.73m** ^**2**^ **)**	69.52 ± 33.65	80.10 ± 28.18	66.87 ± 34.45	**0.032**
**Proteinuria (g/24 h)**	3.50 (1.71, 5.63)	1.53 (0.62, 3.49)	4.08 (2.17, 5.83)	**< 0.001**
PAR				
< 7.155	72	28	44	**< 0.001**
≥7.155	123	11	112	

Bold values indicate statistical significance.

### 3.2 Diagnostic performance and cutoff values for PAR

**Figure 2:**
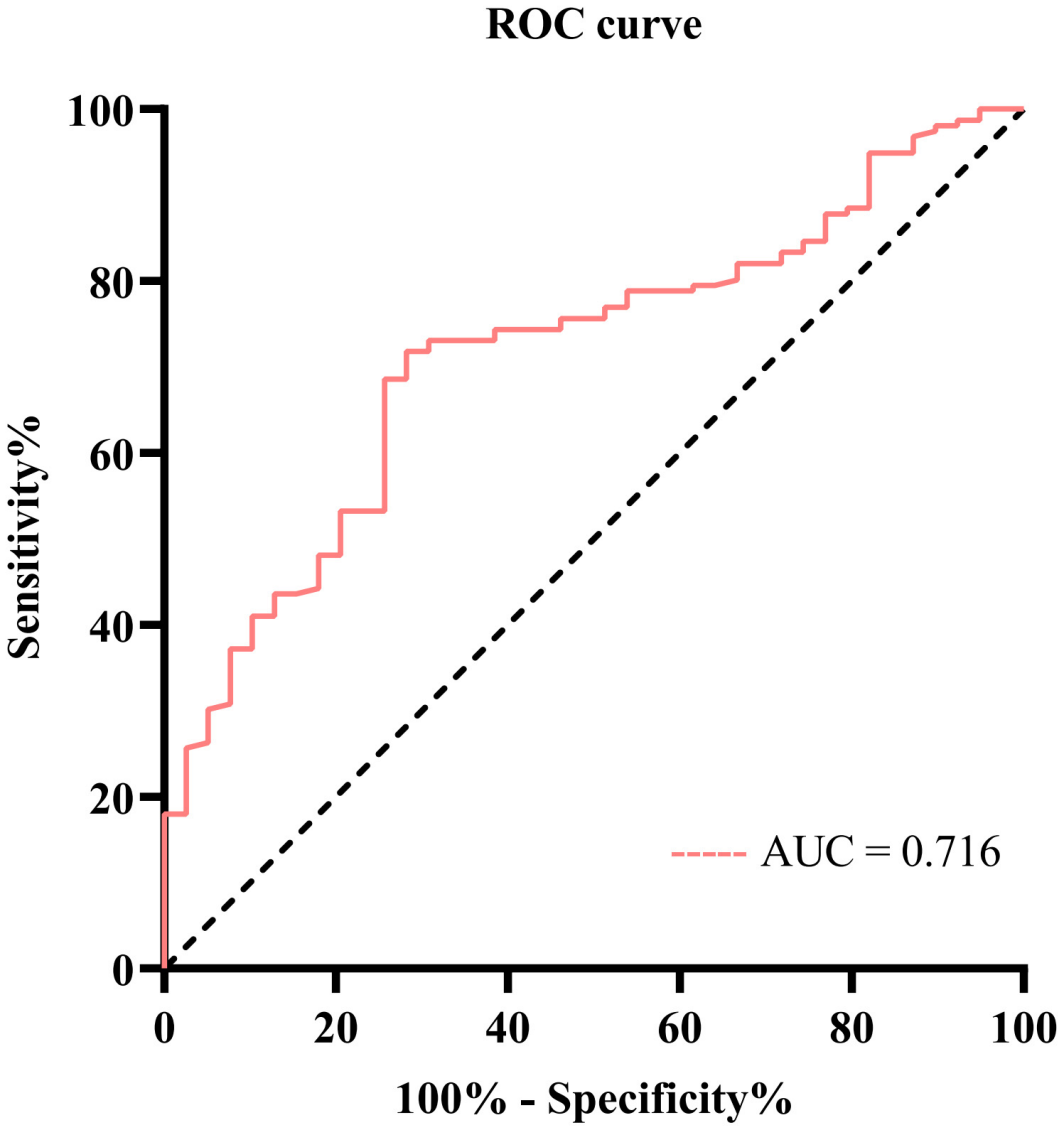
ROC curves for the diagnostic accuracy of the PAR to predict severe DN.

The current study employed ROC curve analysis to assess the diagnostic accuracy of the PAR for severe DN. The results indicated that the AUC for PAR was 0.716 (Figure 2), with a 95% Confidence Interval (CI) (0.634, 0.798), and a significance level of *P* < 0.001. The findings demonstrated that PAR possesses moderate diagnostic accuracy for severe DN, and the results were statistically significant. The optimal cutoff value for PAR was determined to be 7.155, with corresponding sensitivity and specificity values of 0.718. To evaluate the clinical utility of the PAR cutoff (7.155), we constructed a confusion matrix and calculated predictive values. As shown in Supplementary Table S2, the model achieved a Positive Predictive Value (PPV) of 91.1% and a Negative Predictive Value (NPV) of 38.9%. The high PPV suggests that elevated PAR is a strong indicator of severe pathology, whereas the modest NPV indicates that a low PAR value does not reliably exclude the presence of severe DN. These cutoff values effectively balance sensitivity and specificity in predicting severe DN (Table 2). Table 1 displays the baseline clinicopathological characteristics of patients who were divided into two groups according to the optimal PAR cutoff. There were 72 patients in the low PAR group (PAR < 7.155) and 123 patients in the high PAR group (PAR ≥ 7.155). Patients with elevated PAR levels exhibited more severe pathological lesions. These findings suggested a correlation between the PAR index and the pathological lesions associated with DN.

**Table 2:** ROC curves for the diagnostic accuracy of the PAR to predict severe DN.

Indicator	Cutoff	AUC	95% CI	*P-value*	Sensitivity	Specificity
PAR	7.155	0.716	0.634–0.798	< 0.001	0.718	0.718

### 3.3 Multivariate logistic analysis of severe DN

Univariate logistic analysis revealed six baseline clinicopathologic parameters that were significantly associated with the severity of pathological lesions, defined as pathologically confirmed severe DN (*P* < 0.05; Table 1). Based on the clinically prespecified adjustment strategy, a multivariable logistic regression model was constructed including age, sex, hypertension, BMI, CRP, Hb, and eGFR. The results demonstrated that PAR ≥ 7.155 remained an independent predictor of severe DN [adjusted (OR): 6.65; 95% CI: 2.617–16.9; *P* < 0.001] after controlling for these potential confounders (Table 3). When modeled as a continuous variable, PAR remained significantly associated with severe DN (OR: 1.52 per unit increase, 95% CI: 1.18–1.95, *P* = 0.001). The RCS analysis revealed no significant non-linear association between PAR and severe DN risk (*P* for non-linearity > 0.05), supporting the validity of the linear model. To address concerns regarding structural collinearity, we performed additional diagnostics. The correlation matrix (Supplementary Table S3) confirmed strong correlations between PAR and its components. Variance Inflation Factor (VIF) analysis indicated severe multicollinearity when PAR, PLT, and albumin were included simultaneously (VIF > 10 for all; Supplementary Table S4), validating our decision to exclude PLT and albumin from the primary model. Furthermore, a sensitivity analysis was conducted by replacing PAR with PLT and albumin in the multivariable model. In this alternative model, PLT remained an independent predictor (OR: 1.011, 95% CI: 1.001–1.021, *P* = 0.027), while albumin did not show statistical significance (*P* = 0.560; Supplementary Table S5). These findings suggest that the predictive value of PAR for histologic severity of PAR is largely driven by the platelet component, and PAR serves as a robust composite marker.

**Table 3:** Multivariate logistic analysis of severe DN (with PAR as continuous variable).

Variable	β	Wald	*P-value*	Odds ratio	95% CI
Age (years)	−0.009	0.164	0.685	0.991	0.951–1.034
Sex	0.137	0.065	0.798	1.147	0.401–3.278
Hypertension	0.031	0.004	0.947	1.032	0.411–2.587
BMI (kg/m^2^)	−0.036	0.54	0.463	0.964	0.875–1.062
CRP (mg/L)	−0.136	2.638	0.104	0.873	0.74–1.029
Hb (g/L)	−0.014	4.579	0.032	0.986	0.974–0.999
sCr (μmol/L)	0.024	3.519	0.061	1.024	0.999–1.050
eGFR (ml/min/1.73 m^2^)	0.02	1.432	0.232	1.021	0.987–1.055
Proteinuria (g/24 h)	0.275	7.057	0.008	1.316	1.075–1.612
PAR ≥ 7.155	1.895	15.852	< 0.001	6.65	2.617–16.9
PAR (continuous)	0.418	10.71	< 0.001	1.52	1.18–1.95

### 3.4 Fusion LLM performance for glomerular lesion classification

The prediction of glomerular lesion classification (I/II vs. III/IV) serves as a critical initial screening step for DN. The comprehensive comparison results of the proposed fusion LLM against baseline models are presented in the left panel of Table 4. In the binary glomerular lesion classification task, the clinical baseline model achieved a competitive AUC (0.78) but struggled with class imbalance, yielding a lower F1-score (75.22%) and sensitivity (68.15%). In contrast, the Fusion LLM significantly improved the F1-score to 84.92% and sensitivity to 88.15%, demonstrating its advantage in capturing complex relationships beyond linear predictors. In this binary classification task, the RF model achieved a high sensitivity of 91.11%. Although the RF model exhibited a low specificity value of 31.67%, this indicates a significant bias toward the majority class. Furthermore, the RF model predominantly classified patients as severe in order to maximize recall, which consequently resulted in a high false positive rate. In a clinical context, the application of such a model could lead to widespread overdiagnosis and increased anxiety among patients.

**Table 4:** Comprehensive performance comparison for the glomerular lesion classification prediction and pathological IFTA scoring.

Model	Glomerular lesion classification				IFTA Scoring			
	F1-score	AUC	Sensitivity	Specificity	Macro-F1	Macro-AUC	Macro-sensitivity	Macro-specificity
Clinical LR	75.22	**0.78**	68.15	**73.33**	47.74	0.67	49.28	75.74
Logistic regression	76.46	0.73	73.33	**60.00**	41.28	0.59	41.02	72.48
SVM (RBF)	77.29	0.73	75.56	56.67	43.29	0.65	43.54	74.16
Random forest	82.24	0.73	**91.11**	31.67	44.80	**0.72**	46.13	74.80
XGBoost	77.79	0.69	80.00	43.33	45.22	0.67	45.90	74.84
Fusion LLM (proposed model)	**84.92** **±3.84**	**0.75** **±0.06**	88.15 ± 5.93	56.67 ± 6.24	**51.00** **±5.71**	0.64 ± 0.07	**53.73** **±5.68**	**78.06** **±2.93**

The best results are highlighted in bold, and the second best are underlined.

In comparison to all baseline methods, the fusion LLM achieved the highest average F1-score of 84.92 ± 3.84% and an AUC of 0.75 ± 0.06. Additionally, LLM fusion demonstrated a sensitivity of 88.15 ± 5.93% and a specificity of 56.67 ± 6.24%, reflecting a 23.33% improvement over the RF baseline. These findings indicate that by incorporating semantic medical knowledge from LLMs, the proposed framework effectively learns to differentiate between true mild/moderate cases and severe cases. Consequently, it relies on learned distinctions rather than statistical guessing, thereby serving as a more balanced clinical screening tool.

### 3.5 Fusion LLM performance for pathological IFTA scoring

Forecasting pathological IFTA scores, specifically distinguishing among classes 0–1, 2, and 3, presents a significant challenge due to the close similarity among intermediate pathological stages. The multiclass classification results are summarized in the right panel of Table 4. As indicated, traditional machine learning approaches demonstrated limited capability in capturing complex non-linear relationships, with macro-F1 scores ranging from 41.28% to 45.22%. The XGBoost model emerged as the best-performing baseline, achieving a macro-F1 score of 45.22%. In contrast, while the RF model attained a relatively high macro-AUC of 0.72, indicating robust ranking capability, its decision boundaries were less precise, resulting in a lower macro-F1 score compared to XGBoost.

The proposed fusion LLM framework significantly outperformed all baseline models in diagnostic accuracy, achieving a macro-F1 score of 51.00 ± 5.71%. This represents an improvement of 7.05% over the strongest baseline. Furthermore, the proposed model exhibited more balanced performance, with a macro-sensitivity of 53.73% and the highest macro-specificity (78.06%) among all compared models. Although the macro-AUC for the proposed model (0.64) was slightly lower than that of the RF model, the substantial increase in F1-score indicates that the proposed model is more decisive and accurate in generating final categorical predictions. This advantage was particularly evident for the intermediate class, which is the most challenging to differentiate in practice. These results underscore the effectiveness of the tabular-to-text strategy in capturing fine-grained pathological features that are not adequately represented by numerical models. To further verify the training stability and rule out severe overfitting, we analyzed the learning curves derived from the 5-fold cross-validation process. As illustrated in Supplementary Figures S1 and S2, the validation F1-scores rapidly converged and maintained a stable plateau without exhibiting the catastrophic degradation typical of severe data memorization. This indicates that our aggressive regularization strategies effectively constrained the hypothesis space.

### 3.6 Incremental predictive value of PAR

To evaluate whether PAR adds incremental discriminative value beyond established clinical variables, we compared the performance of a baseline clinical model against a PAR-enhanced model. The baseline model, which included age, sex, eGFR, hemoglobin, and 24-h proteinuria, achieved an AUC of 0.762 (95% CI: 0.673–0.851). In contrast, the PAR-enhanced model demonstrated superior discriminative ability with an AUC of 0.828 (95% CI: 0.748–0.908). The improvement in the area under the curve was statistically significant (ΔAUC = 0.066, *P* < 0.01, DeLong test). Furthermore, the Integrated Discrimination Improvement (IDI) was 0.082 (*P* < 0.01), indicating a significant enhancement in the model's reclassification ability. These results suggest that PAR provides significant incremental value in predicting severe DN beyond standard clinical parameters (Supplementary Table S6).

## 4 Discussion

This analysis demonstrates a significant association between the PAR and the pathological severity of DN. Additionally, a “tabular-to-text-to-vector” framework was introduced to leverage LLMs without the need for fine-tuning for non-invasive DN grading. The present analysis indicates that a PAR ≥ 7.155 serves as an independent risk factor for severe glomerular lesions (grades III–IV), with an AUC of 0.716 indicating moderate diagnostic accuracy. Moreover, the fusion LLM framework outperformed the multilayered machine learning baselines in predicting glomerular lesion classification and IFTA scores. It also demonstrated improved sensitivity and specificity.

Based on current findings, a high PAR is significantly associated with severe diabetic nephropathy, consistent with the emerging evidence demonstrating the role of inflammation and malnutrition in the progression of diabetic kidney disease (34, 35). In addition to hemostasis, PLTs also contribute to immune and inflammatory responses. Elevated levels of PLTs are often indicative of a pro-inflammatory and hypercoagulable state, which can exacerbate glomerular endothelial injury (36). Conversely, low albumin levels in patients with DN often indicate malnutrition or severe proteinuria, both of which are recognized contributors to renal decline (37, 38). In IgA nephropathy, the PAR was independently associated with adverse renal outcomes, and a cutoff value of 13.73 was determined to be optimal for identifying high-risk patients (39). The high value of PAR (>4.67) in critically ill patients also had a significant association with 30-day mortality (40).

While the biological rationale supports the utility of PAR, we must strictly evaluate its diagnostic performance. We acknowledge that the AUC of 0.716 indicates moderate, rather than high, discriminative ability. The data-driven cutoff (Youden index) may be optimistic due to the lack of external validation. The analysis of predictive values revealed a dual nature: the high PPV (91.1%) suggests that PAR is effective in identifying patients highly likely to have severe lesions, making it a useful “rule-in” screening tool in resource-limited settings. However, the low NPV (38.9%) is a critical limitation, indicating that 61.1% of patients with a “low risk” PAR value actually had severe pathology (III–IV). Furthermore, our study demonstrated that PAR provides significant incremental discriminative value beyond established clinical variables. The addition of PAR to a baseline model comprising age, sex, eGFR, hemoglobin, and 24-h proteinuria resulted in a significant improvement in the AUC (from 0.762 to 0.828, *P* < 0.01) and a positive Integrated Discrimination Improvement (IDI = 0.082, *P* < 0.01). This indicates that PAR captures prognostic information not fully represented by traditional clinical parameters. While eGFR and proteinuria are essential indicators of renal function, PAR may reflect underlying systemic inflammation and nutritional status, which are critical pathological mechanisms in the progression of diabetic nephropathy. Therefore, incorporating PAR into clinical assessment models could enhance the accuracy of risk stratification for patients with severe DN, potentially guiding more personalized therapeutic strategies. Consequently, PAR should not be used to defer renal biopsy in patients with clinically suspected severe DN, but rather repositioned as an exploratory biomarker for risk stratification to assist in prioritizing high-risk cases. Despite this, the PAR presents a pragmatic and cost-effective option for clinicians to identify patients at risk for severe pathology, and the integration of machine learning (ML) with PAR analysis offers unprecedented opportunities for DN risk stratification.

According to researchers, traditional ML models, particularly interpretable ones such as Random Forest, demonstrate higher accuracy in predicting the progression of DN (41, 42). Notably, the RF algorithm identifies several key predictors, including the neutrophil-to-lymphocyte ratio (43), while algorithms such as XGBoost achieve high AUC values using extensive features. Deep learning models frequently face challenges associated with limited medical data, particularly due to the difficulties involved in collecting large-scale invasive biopsy samples. However, the proposed fusion LLM framework demonstrated robustness despite the small sample size (*n* = 195). A critical limitation was observed in traditional machine learning models; for instance, the Random Forest algorithm exhibited a sensitivity-specificity trade-off, achieving high sensitivity (91.11%) but low specificity (31.67%). To address these issues, the proposed framework employs a “tabular-to-text” strategy for latent pattern extraction, utilizing a weighted cross-entropy loss alongside features derived from the frozen Qwen and Llama models. Crucially, this “no-fine-tuning” paradigm mitigates the risks of hallucination and reduces the high computational costs typically associated with generative AI. Consequently, the proposed model achieved a balanced specificity of 56.67 ± 6.24% and an F1 score of 84.92% in the glomerular lesion classification task. In the IFTA multi-classification task, the proposed model significantly surpassed the performance of XGBoost, achieving a Macro-F1 score of 51.00 ± 5.71%, compared to 45.22%.

The strengths of this study include the utilization of biopsy-verified real data, strict inclusion criteria, and robust statistical validation. Additionally, the biological plausibility of PAR as a marker for both inflammatory and metabolic pathways further reinforces these findings (39). However, several limitations should be acknowledged. First, due to the lack of longitudinal follow-up data, no conclusions can be drawn regarding the prognostic value of PAR for renal endpoints; thus, our findings should be regarded as hypothesis-generating for future prospective studies. Second, the single-center design and reliance on a biopsy-proven cohort may limit the generalizability of our results to the broader diabetic population. The “biopsy-selected” nature introduces potential spectrum bias, as patients undergoing biopsy often present with atypical clinical courses or suspected non-diabetic kidney disease, which may not represent the typical natural history of DN. Third, the exclusion of patients with an eGFR < 30 mL/min/1.73 m^2^ restricts the applicability of our findings to patients with early-to-moderate chronic kidney disease (CKD stages 1-3), as those with advanced renal failure (CKD stages 4–5) were not included. Finally, we must recognize the fundamental limitation concerning dimensionality vs. sample size.

Although our co-occurrence strategy, data augmentation, and heavy regularization effectively mitigated empirical performance degradation, we carefully evaluated the feasibility of performing formal statistical tests (e.g., McNemar's or DeLong's test) between models. Given our cohort size (*n* = 195) and 5-fold cross-validation approach, validation sets per fold are small (~39 samples), limiting the statistical power for non-parametric tests. Pooling predictions across folds violates the independence assumption. Therefore, we relied on consistent performance margins (e.g., +7%−10% in F1) and stable cross-validation variance, evidenced by the learning curves, to demonstrate the reliability of our model comparisons. Training a network with approximately 46.5 million parameters on a dataset of *N* = 195 patients carries an inherent risk of overfitting. Therefore, this study should be strictly viewed as an exploratory proof-of-concept demonstrating the feasibility of employing fine-tuning-free LLM representations for small-sample nephrology tasks. Future multicenter studies incorporating non-biopsy populations and late-stage CKD patients are essential to validate the clinical utility of PAR and the fusion LLM framework across the full disease spectrum, optimize explainable AI tools, and extend this framework to encompass multimodal analyses. A major limitation of this study is the lack of data regarding the specific use of RAAS inhibitors (ACEIs/ARBs). Given that RAAS blockers are the preferred antihypertensive therapy for patients with DN and directly influence proteinuria and serum albumin levels, the inability to adjust for this variable introduces a significant risk of residual confounding. Consequently, we cannot draw definitive conclusions regarding the independent association between PAR and DN pathological severity. Future prospective studies must incorporate detailed medication histories to control for this critical confounder and validate our exploratory findings.

In conclusion, the integration of PAR and LLM-based analytics presents a promising avenue for advancing DN management by bridging the mechanistic relevance of biomarkers with the predictive power of artificial intelligence. This research identifies PAR as a significant marker for DN severity and proposes a fine-tuning-free fusion LLM framework as an exploratory proof-of-concept for non-invasive risk stratification. By addressing critical challenges such as small sample sizes and limited computational resources, this approach offers a potential adjunctive strategy to facilitate early decision-making for high-risk patients and guide personalized therapies targeting inflammation and metabolic derangement. However, given the modest NPV, lack of external validation, and the unavailability of RAAS inhibitor usage data—a major confounding factor—this study cannot draw definitive conclusions regarding the independent association of PAR. Thus, it should be regarded as an exploratory hypothesis-generating study, underscoring the need for further prospective validation with comprehensive medication records.

## Data Availability

The datasets presented in this study can be found in online repositories. The names of the repository/repositories and accession number(s) can be found in the article/Supplementary material.

## Supplementary material

Supplementary Figure S1 Learning curves for glomerular lesion classification (Binary).

Supplementary Figure S2 Learning curves for IFTA scoring (Multiclass).

Supplementary Table S1 Data conversion logic and example.

Supplementary Table S2 Confusion matrix and predictive values for PAR (Cutoff = 7.155).

Supplementary Table S3 Correlation matrix of key variables.

Supplementary Table S4 Collinearity diagnostics using Variance Inflation Factor (VIF).

Supplementary Table S5 Sensitivity analysis multivariable logistic regression model (Replacing PAR with PLT and Albumin).

Supplementary Table S6 Incremental predictive value of PAR for severe diabetic nephropathy.
