# Supplementary figures and images for: Association of the platelet-to-albumin ratio with diabetic nephropathy lesions via a fine-tuning-free large language model framework
Source: Front Med (Lausanne). 2026 May 20;13:1793422. doi: 10.3389/fmed.2026.1793422 (PMC13229808; doi:10.3389/fmed.2026.1793422)

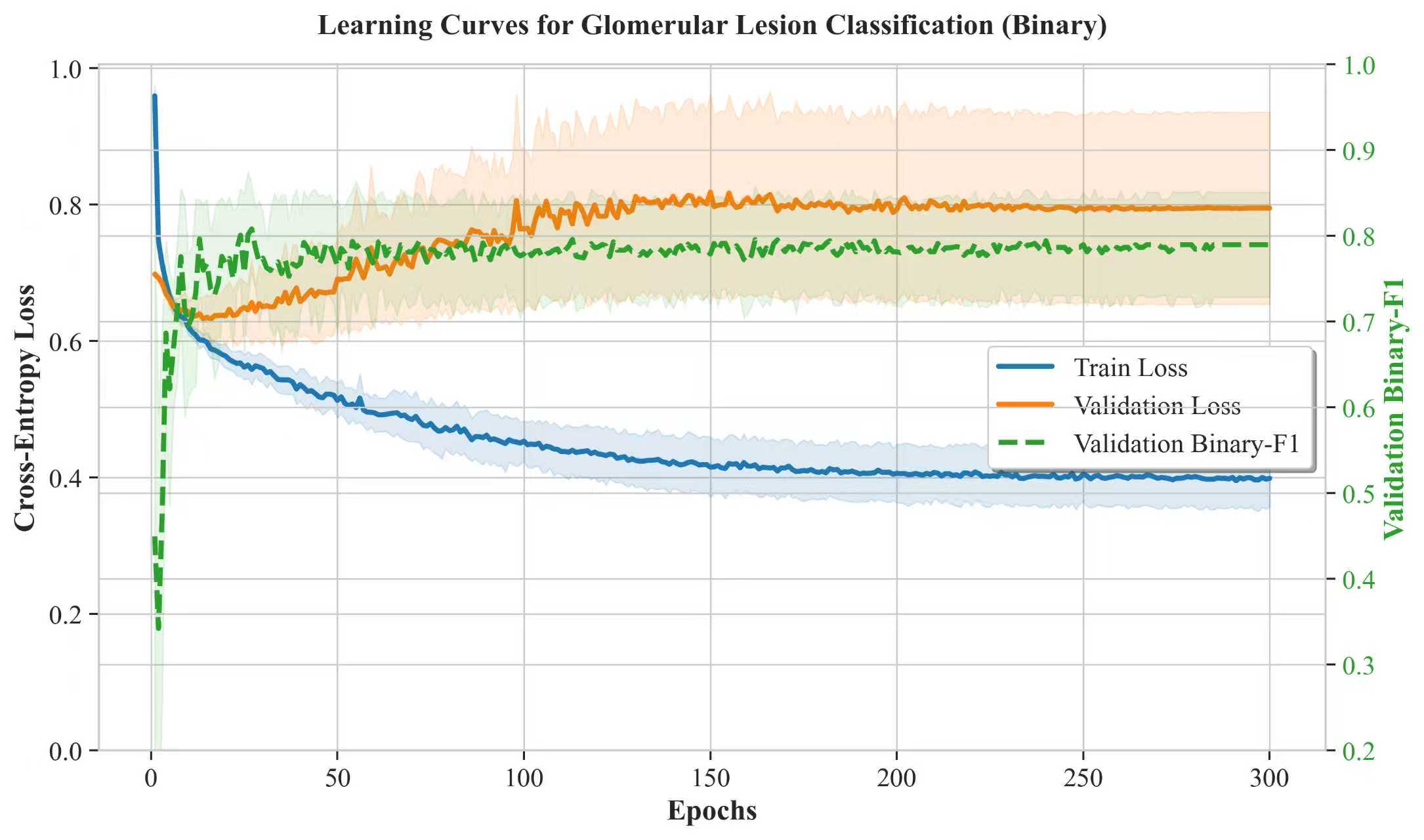

Supplement: Supplementary Figure S1 — Learning curves for glomerular lesion classification (Binary). [file Image_1.jpeg]

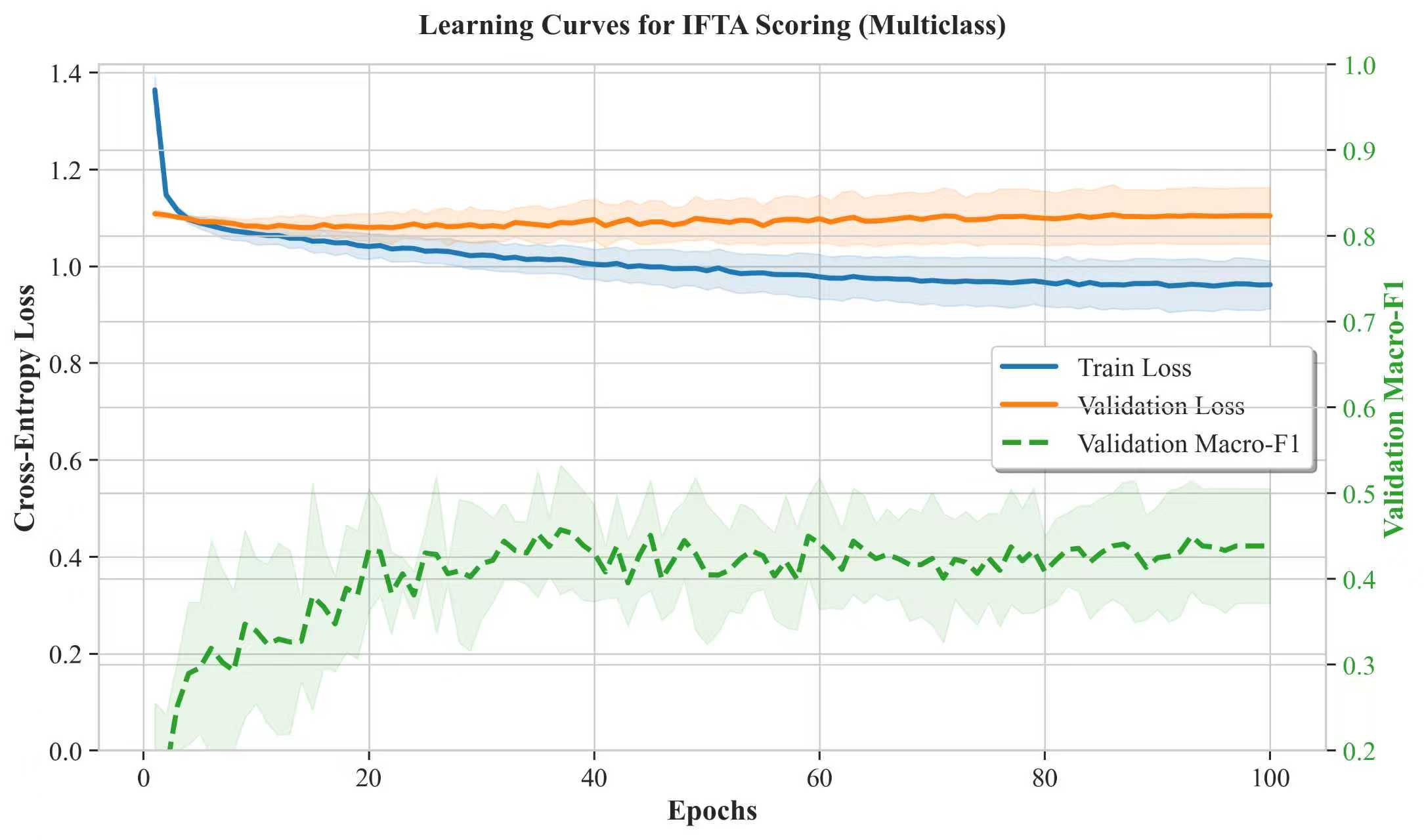

Supplement: Supplementary Figure S2 — Learning curves for IFTA scoring (Multiclass). [file Image_2.jpeg]
