# Supplementary material for: Association of the platelet-to-albumin ratio with diabetic nephropathy lesions via a fine-tuning-free large language model framework
Source: Front Med (Lausanne). 2026 May 20;13:1793422. doi: 10.3389/fmed.2026.1793422 (PMC13229808; doi:10.3389/fmed.2026.1793422)
Supplement: Supplementary Table S1 — Data conversion logic and example. [file Table_1.docx]

**Supplementary Table S1. Data Conversion Logic and Example.**

| **Section** | **Raw Data (Tabular)** | **Generated Text Segment** | **Methodological Note** |
| --- | --- | --- | --- |
| Demographics | Age:58 Gender:1 History:10 | "Basic Information:58 years old Male,history of diabetes for 10 years. ” | Gender mapped (1→Male). Age rounded to integer. |
| Physical Exam | BMI:26.5 BP:152/95 HTN:1(Yes) | "Physical Examination:BMI 26.5, Comorbid Hypertension, BP 152/95mmHg." | Logic Injection: lExplicitly adds "Co- morbid Hypertension” context if binary flag is present. |
| Renal Function | eGFR:55.2 SCr:110.5 Pro:850.5 | "Renal Function:eGFR is 55.2 ml/min.24-hour proteinuria severity is 850.500 mg… ” | Precision Control: Proteinuria retains 3 decimal places to capture micro-variations. |
| Core Indicator | PAR:6.2 | "Core Indicator: Platelet-to- Albumin Ratio(PAR)is 6.200.” | Highlighted as the pri- mary variable of interest. |
| Metabolic Context | Glu:6.8 Hb:125 CRP:3.2 | "Metabolic &Hematologic Con- text: Glucose 6.80 mmol/L.. Hemoglobin 125 g/L… ” | Contextual grouping of inflammatory and metabolic markers. |

Supplementary Table S2. Confusion Matrix and Predictive Values for PAR (Cutoff = 7.155).

|  | **Pathologically Severe DN (III-IV)** | **Pathologically Mild DN (I-II)** | **Total** |
| --- | --- | --- | --- |
| PAR ≥ 7.155 (Positive) | 112 (True Positive) | 11 (False Positive) | 123 |
| PAR < 7.155 (Negative) | 44 (False Negative) | 28 (True Negative) | 72 |
| Total | 156 | 39 | 195 |
| Positive Predictive Value (PPV) |  |  | 91.1% |
| Negative Predictive Value (NPV) |  |  | 38.9% |
| Sensitivity |  |  | 71.8% |
| Specificity |  |  | 71.8% |

Supplementary Table S3. Correlation matrix of key variables.

| **Variable** | **PAR** | **Platelet (PLT)** | **Albumin (ALB)** | **Proteinuria** |
| --- | --- | --- | --- | --- |
| PAR | 1.000 | 0.541* | -0.694* | 0.207* |
|  |  | (<0.001) | (<0.001) | (0.004)* |
| Platelet (PLT) |  | 1.000 | -0.054 | 0.067 |
|  |  |  | (0.451) | (0.349) |
| Albumin (ALB) |  |  | 1.000 | -0.327* |
|  |  |  |  | (<0.001) |
| Proteinuria |  |  |  | 1.000 |

Note:* indicates P < 0.05.

Supplementary Table S4. Collinearity diagnostics using Variance Inflation Factor (VIF).

| **Variable** | **B** | **Std. Error** | **Beta** | **t** | **P** | **Tolerance** | **VIF** |
| --- | --- | --- | --- | --- | --- | --- | --- |
| PLT (10^9^/L) | 0.001 | 0.002 | 0.088 | 0.291 | 0.772 | 0.049 | 20.272 |
| ALB (g/L) | 0.001 | 0.02 | 0.014 | 0.051 | 0.959 | 0.061 | 16.525 |
| Proteinuria | 0.029 | 0.008 | 0.246 | 3.487 | 0.001 | 0.906 | 1.103 |
| PAR | 0.045 | 0.071 | 0.212 | 0.638 | 0.524 | 0.041 | 24.597 |

Note: VIF > 10 indicates severe multicollinearity.

Supplementary Table S5. Sensitivity analysis multivariable logistic regression model (Replacing PAR with PLT and Albumin).

| **Variable** | **B** | **S.E.** | **Wald** | **P value** | **Odds Ratio (OR)** | **95% CI for OR** |
| --- | --- | --- | --- | --- | --- | --- |
| Age (years) | -0.008 | 0.015 | 0.28 | 0.596 | 0.992 | 0.963 – 1.022 |
| Sex (Male) | 0.28 | 0.43 | 0.42 | 0.517 | 1.323 | 0.569 – 3.078 |
| Hypertension | 0.25 | 0.42 | 0.35 | 0.554 | 1.284 | 0.563 – 2.931 |
| BMI (kg/m²) | -0.03 | 0.05 | 0.36 | 0.548 | 0.970 | 0.881 – 1.069 |
| CRP (mg/L) | -0.04 | 0.04 | 1.12 | 0.290 | 0.961 | 0.891 – 1.036 |
| Hb (g/L) | -0.015 | 0.007 | 4.52 | 0.034 | 0.985 | 0.971 – 0.999 |
| eGFR | 0.004 | 0.008 | 0.25 | 0.617 | 1.004 | 0.988 – 1.020 |
| Proteinuria (g/24 h) | 0.268 | 0.092 | 8.51 | 0.004 | 1.308 | 1.091 – 1.568 |
| PLT (10⁹/L) | 0.011 | 0.005 | 4.86 | 0.027 | 1.011 | 1.001 – 1.021 |
| Albumin (g/L) | -0.025 | 0.043 | 0.34 | 0.560 | 0.975 | 0.896 – 1.061 |
| Constant | -0.85 | 1.75 | 0.24 | 0.626 | 0.427 |  |

Supplementary Table S6. Incremental predictive value of PAR for severe diabetic nephropathy.

| **Model** | **Variables Included** | **AUC (95% CI)** | **P Value vs. Baseline** | **IDI (95% CI)** | **P Value for IDI** |
| --- | --- | --- | --- | --- | --- |
| Baseline Model | Age, Sex, eGFR, Hb, Proteinuria | 0.762 (0.673–0.851) | — | — | — |
| PAR-Enhanced Model | Baseline + PAR | 0.828 (0.748–0.908) | <0.01 | 0.082 | <0.01 |

Note: AUC = Area Under the Curve; CI = Confidence Interval; IDI = Integrated Discrimination Improvement.
